# α-Ketoglutarate Ameliorates Sarcopenia in D-Galactose-Induced Aging Mice by Modulating Protein Homeostasis and Optimizing Mitochondrial Function

**DOI:** 10.3390/nu17213336

**Published:** 2025-10-23

**Authors:** Yangguang Zhang, Huihui Wang, Yijia Zhang, Xintong Wang, Ziyu Qiao, Jiayu Wang, Yixuan Li, Yanan Sun

**Affiliations:** 1 Beijing Advanced Innovation Center for Food Nutrition and Human Health, Department of Nutrition and Health, China Agricultural University, Beijing 100193, Chinaxtwang@cau.edu.cn (X.W.);; 2 State Key Laboratory of Food Nutrition and Safety, College of Food Science and Engineering, Tianjin University of Science and Technology, Tianjin 300457, China; 3 Key Laboratory of Functional Dairy, Co-Constructed by Ministry of Education and Beijing Municipality, College of Food Science & Nutritional Engineering, China Agricultural University, Beijing 100083, China

**Keywords:** α-ketoglutarate, sarcopenia, protein homeostasis, mitochondrial function, antioxidation

## Abstract

**Background/Objectives:** Sarcopenia is an age-related condition marked by a progressive decline in muscle mass, weakened strength, and decreased physical performance in the elderly. **Methods:** In this research, we used D-galactose (D-gal)-induced 8-week-old male C57BL/6J mice to establish a sarcopenia model. This model was utilized to investigate the effect and potential mechanism of α-ketoglutaric acid (AKG), a key intermediate of the tricarboxylic acid cycle, on sarcopenia. **Results:** Our findings demonstrated that AKG significantly ameliorated muscle mass, exercise endurance, grip strength, and cold tolerance in D-gal-induced aging mice. AKG could regulate protein homeostasis, thereby enhancing the protein composition and size of myofibers in D-gal-induced aging mice. Additionally, AKG enhanced SOD activity in the skeletal muscle of D-gal-induced aging mice and scavenged reactive oxygen species (ROS) by activating the SIRT1/PGC-1α/Nrf2 pathway, thereby improving mitochondrial function. **Conclusions:** In conclusion, AKG combated sarcopenia by regulating protein homeostasis and optimizing mitochondrial function in skeletal muscle. This study provides a scientific foundation for developing therapeutic interventions using AKG to target muscle aging.

## 1. Introduction

Skeletal muscle is important in maintaining mobility, metabolic homeostasis, and overall physical health, making its deterioration a significant health concern [1,2]. Sarcopenia is a disease marked by progressive decline in muscle mass [3], strength, and function, which severely impairs quality of life and independence, especially in the elderly [4,5]. Epidemiological studies indicate that past 50 years, muscle mass decreases by 1% to 2% every year [6], while muscle strength diminishes at an even faster rate of 1.5–3% per year [7]. As global population aging intensifies, the pathological mechanisms and intervention strategies for sarcopenia have become important directions in geriatric medicine and metabolic research [8]. Currently, clinical interventions for sarcopenia mainly include nutritional supplements (such as proteins and vitamin D), resistance training, and hormone replacement therapy (such as testosterone and growth hormone) [9]. However, existing strategies have significant limitations: nutritional supplementation has limited effects on patients with severe sarcopenia, and excessive protein intake may worsen kidney burdens; while exercise interventions can improve muscle function, adherence is low among elderly individuals with mobility issues or coexisting chronic diseases [10]. Hormone therapy is associated with side effects such as cardiovascular risks and prostate enlargement, which limits its long-term use [11]. Therefore, the exploration of new, safe, and effective intervention targets is urgently needed.

Due to their accessibility and safety, dietary interventions have gained attention as a viable strategy for managing sarcopenia. Among potential candidates, α-ketoglutarate, a key metabolic intermediate in the TCA cycle, has shown promise [12,13]. AKG is involved in cellular energy metabolism, nitrogen balance, and antioxidant defense mechanisms [14]. Furthermore, AKG can be naturally produced in mice and humans, and regulatory agencies have recognized it is safe and edible [15]. Previous studies have demonstrated its beneficial effects in skeletal muscle [14]. Compared with young mice, the level of AKG in primary myoblasts of aged mice was significantly lower, and supplementing AKG could promote the proliferation of myoblasts in aged mice [16]. Ciuffoli et al. (2024) found that injecting AKG on days 0, 1, 3, and 5 after skeletal muscle injury could significantly increase the number of muscle stem cells and the cross-sectional area of regenerated muscle fibers [16]. Cai et al. (2018) discovered that compared with control mice, supplementing AKG (2% of body weight) could effectively improve the skeletal muscle strength, endurance, weight, gastrocnemius muscle weight and peroneal muscle weight of Mdx mice [17]. Furthermore, Asadi et al. demonstrates that by supplementing AKG in the form of calcium salt (CaAKG) through diet, the levels of inflammatory cytokines throughout the body were reduced, while alleviating the state of weakness and promoting a longer and healthier lifespan [18]. Although the beneficial effects of AKG on skeletal muscle mass and function are evident, there are still many gaps in current research. Most studies come from pre-clinical research or small-scale clinical trials, and the potential mechanism by which AKG functions remains unclear.

In this study, we established a skeletal muscle atrophy model induced by D-galactose (D-gal) to induce oxidative stress and inflammation, systematically evaluating the therapeutic potential of AKG on skeletal muscle mass, function, and mitochondrial function, with a particular focus on its underlying molecular mechanisms. The results of this study are expected to provide new insights for the application of AKG in muscle enhancement and to serve as a foundation for developing intervention measures for muscle atrophy diseases.

## 2. Materials and Methods

### 2.1. Main Reagents

α-Ketoglutaric acid (AKG, C16111850, Northridge, CA, USA) was purchased from Macklin. D-galactose (D-gal, G5388) was purchased from Sigma-Aldrich (St. Louis, MO, USA). Antibodies used were Phospho-Akt (Ser473) Antibody (Cell Signaling, 9271, Danvers, MA, USA), Akt Antibody (Cell Signaling, 9272), Phospho-mTOR (Ser2448) Antibody (Cell Signaling, 2971), mTOR (Cell Signaling, 2972), MuRF1 (Santa Cruz Biotechnology, sc-398608, Dallas, TX, USA), Fbx32 (Abcam, ab168372, Cambridge, UK), SirT1 (D1D7) Rabbit mAb (Cell Signaling, 9475), PGC1α Monoclonal antibody (Proteintech, 66369-1-Ig, Wuhan, China), NRF2 Rabbit mAb (ABclonal, A21176, Wuhan, China), GAPDH polyclonal antibody (Proteintech, 10494-1-AP), HRP-labeled Goat Anti-Rabbit IgG(H + L) (Beyotime, A0208, Shanghai, China), HRP-labeled Goat Anti-Mouse IgG(H + L) (Beyotime, A0216). RIPA Lysis Buffer (Strong, P0013B) was aquired from Beyotime (Shanghai, China). The BCA Protein Assay Kit (PC0020) was acquired from Solarbio (Beijing, China). The cDNA synthesis kit (G592) was purchased from Applied Biological Materials Inc. (abm, Richmond, BC, Canada). The IL-6 (Interleukin-6) ELISA kit (MK5737A)) and TNF-α (Tumor necrosis factor-α) (MK2868A) were purchased from Jiangsu Sumeike Biological Technology Co., Ltd. (Yancheng, China). The ROS ELISA kit (MK6027A) was purchased from mkbio.

### 2.2. Mice

All animals were purchased from Beijing Huafu Kang Biotechnology Co., Ltd. (Beijing, China). During the experiment, all animals were healthy and not genetically modified. The sample size was determined based on preliminary experiments and relevant literature. We obtained 36 male C57BL/6J mice, aged 8 weeks (weighing 25 ± 2 g), and the experiment was conducted at the Animal Experiment Center of the West Campus of China Agricultural University. The rodents were housed in a controlled environment with temperature and humidity steadily held at 22 ± 2 °C and 55 ± 10%, respectively. The lighting regimen in the animal facility was set to switch between light and dark every twelve hours. Weekly measurements of body weight and food intake were taken. Furthermore, all animal care and experimental procedures were reviewed and approved by the Ethics Committee for Animal Experiments of China Agricultural University (Approval No. AW11304202-5-1).

### 2.3. Animal Experiment Design

We used six cages, with six mice in each cage. All mice were assigned ear tags numbered from 1 to 36, and after a one-week acclimation period, they were randomly allocated to control group, model group (D-gal group), and model + intervention group (D-gal + AKG group) using a computer-generated randomization sequence. After grouping, the body weights of the mice were measured to ensure that there were no statistically significant differences in baseline body weight between the groups (less than 0.2 g). A sarcopenia model was established through subcutaneous injections of D-galactose at a dosage of 500 mg/kg daily for a duration of 8 weeks [19]. The control group received an equivalent volume of 0.9% saline. In addition, the D-gal + AKG group received subcutaneous injections of AKG at a dosage of 1 mg/kg/day following the administration of D-gal. At the same time, the animal cages were randomly rotated to minimize bias. A blinded investigator performed subcutaneous injections on the animals every morning between 9 AM and 11 AM. After the experiment, the mice were fasted overnight without food but with access to water for more than 12 h. Blood was collected via retro-orbital puncture, and all mice were euthanized by CO_2_ asphyxiation.

### 2.4. Body Composition

Upon the completion of the eight-week experimental period, the lean muscle mass and fat content of the mice were quantitatively assessed utilizing a small animal awake body composition analyzer [20]. The body weight of each mouse was meticulously recorded prior to the initiation of testing. Subsequently, the mice were placed into the instrument for analysis.

### 2.5. Muscle Endurance Test

A small animal treadmill (Zhongshidichuang, Beijing, China) was used to assess mice’s muscle endurance. The mice were acclimatized for 3 days. On the fourth day, they were placed on the treadmill, with the initial running speed of 6 m/min (increased by 1 m/min every 5 min). The session ended when the mice could not re-mount the treadmill or remained or stayed on the shock grid for 10 s. The total running duration was recorded to evaluate their muscle endurance [21].

### 2.6. Maximal Running Speed Test (Muscle Explosiveness)

The mice were set on a treadmill with an initial speed set of 6 m/min, which was subsequently increased by 5 m/min every 2 min until a maximum speed of 40 m/min was reached. The running session concluded when the mice were unable to get back on the treadmill or remained on the shock grid for 10 s. The speed they was recorded to assess their maximum running capacity.

### 2.7. Grip Strength Test

A grip strength tester (Zhongshidichuang, Beijing, China) was employed to assess muscle strength, positioned horizontally on a flat surface [21]. The mouse was placed on the grip sensing platform, when all four limbs grasped the grip bar, it was pulled horizontally backward until all limbs disengaged from the bar. The maximum grip strength value was automatically recorded by the device. This procedure was conducted three times for each mouse, and the average grip strength was calculated in grams (g).

### 2.8. Body Temperature

A single mouse was housed individually in a cage and subjected to acute cold exposure at 4 °C for a duration of 1 h. Following this exposure, its body surface temperature was immediately measured using a thermal imager, in accordance with the methodology described by Chen et al. [22].

### 2.9. H&E Staining

The tibialis anterior (TA) muscle was fixed in 4% paraformaldehyde/PBS at 4 °C for one week, followed by replacement with 70% ethanol and maintained at 4 °C for at least overnight. After paraffin embedding, sections were cut at the maximum cross-sectional area and placed on slides. Dewaxed sections were stained with regular hematoxylin and eosin (HE). Five representative regions were randomly selected from each sample, analyzing approximately 300 muscle fibers, and the mean cross-sectional area of the muscle myofibers was calculated using 2025 imagej 2 fiji 1.54p software.

### 2.10. Transmission Electron Micrographs (TEMs)

The tibialis anterior muscle samples were fixed in 2.5% glutaraldehyde solution at 4 °C for over 24 h. Following fixation, the tissues underwent gradient dehydration and infiltration treatment. After 4–5 h, the samples were transferred to pure embedding medium and left at room temperature overnight. Once the embedding medium polymerized, the blocks were trimmed and prepared for ultra-thin sectioning. Finally, the ultrastructure of the muscle fibers and the morphology of the mitochondria were observed and imaged using transmission electron microscopy.

### 2.11. Gene Expression Detection

Total RNA was extracted using the TRIzol method. The extracted RNA was then subjected to cDNA synthesis using a cDNA synthesis kit, following the manufacturer’s instructions. Subsequently, qRT-PCR was performed using PowerUp™ SYBR™ Green kit. The relative expression of target genes was normalized to the housekeeping gene GAPDH. GAPDH expression levels are generally stable in the same type of cells or tissues and are minimally affected by external inducers, such as hypoxia or diabetes. Additionally, based on references to other studies related to skeletal muscle [1,23,24], we have chosen GAPDH as the housekeeping gene and protein. The primers used in this study are shown in Table 1.

### 2.12. Western Blot Analysis

Take approximately 50 mg of TA muscle and place it in a 1.5 mL homogenization tube, adding 4–5 glass beads. Next, add 500 μL of RIPA buffer (containing 100 mM PMSF) to the tube. Then, quickly cool the tube in liquid nitrogen and place it in a tissue homogenizer to grind thoroughly. After homogenization, centrifuge the mixture at 4 °C for 15 min (12,000× *g*) and collect the supernatant. Use the BCA Protein Assay Kit to determine the protein concentration. The protein samples were mixed with a 5x sample loading buffer in a ratio of 4:1 (*v*/*v*). After boiling at 95 °C for 5 min, the samples were placed at −80 °C. The Western Blot assay was conducted using 4% concentrated gel, 10% or 12% separating gel, and a sample size of 30 μg [24].

### 2.13. ROS Measurements

The obtained blood was left at room temperature for approximately two hours. The supernatant was obtained after centrifugation at 4 °C for 15 min. They were then dispensed in 0.5 mL sterile enzyme-free centrifuge tubes and transferred to −80 °C for storage. ROS in serum was measured according to the ELISA kit instructions.

### 2.14. MDA Measurements

Collected blood sat at room temperature for about 2 h. The liquid was obtained by centrifugation at 4 °C for 15 min and then aliquots were placed in sterile 0.5 mL enzyme-free centrifuge tubes. 0.1 mL of varying concentration standards was added to create the standard curve, and 0.1 mL of samples were added to the assay. Finally, the absorbance was determined at 532 nm by use of a microplate reader.

### 2.15. Inflammatory Factors Measurements

The obtained blood was centrifuged at room temperature for about 2 h before collecting the supernatant, which was then aliquots in 0.5 mL sterile enzyme-free centrifuge tubes. According to the ELISA kit instructions, the inflammatory factors (TNF-α and IL-6) in serum were measured.

### 2.16. Statistical Analysis

All findings are presented in the form of average plus or minus SEM. Statistical significance was determined by one-way ANOVA with Tukey post hoc tests for multiple-group comparisons. All figures were plotted with GraphPad Prism 8.

## 3. Results

### 3.1. Effect of AKG on Skeletal Muscle Mass and Function in D-Gal-Induced Aging Mice

**Figure 1:**
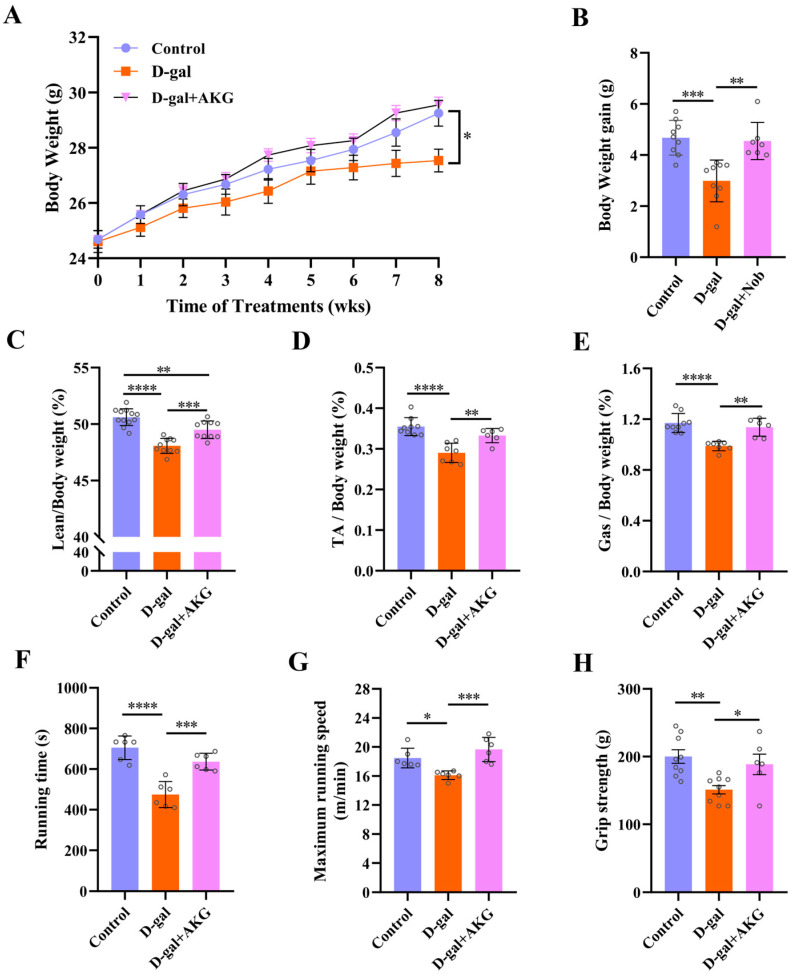
Effects of AKG on muscle mass and exercise capacity in D-gal-induced aging mice. (**A**) Body weight, (*n* = 6~12 mice/group). (**B**) Body weight gain, (*n* = 6~12 mice/group). (**C**) Lean mass to body weight ratio, (*n* = 6~12 mice/group). (**D**) Tibialis (TA) mass to body weight ratio, (*n* = 6~12 mice/group). (**E**) Gastrocnemius (Gas) mass to body weight ratio, (*n* = 6~12 mice/group). (**F**) Running time, (*n* = 6 mice/group). (**G**) Maximum running speed, (*n* = 6 mice/group). (**H**) Grip strength, (*n* = 6 mice/group). * *p* < 0.05, ** *p* < 0.01, *** *p* < 0.001, **** *p* < 0.0001.

To investigate the skeletal muscle-enhancing effects of AKG, we employed D-gal-induced aging model to establish skeletal muscle atrophy [25]. During the entire experiment, the body weight of all mice was monitored (Figure 1A). After 8 weeks of injection, the body weight of mice in the model group decreased by 6.1% relative to the control group. AKG treatment resulted in a 7.3% increase in body weight in D-gal-induced aging mice compared to the model group (Figure 1B). Furthermore, through body composition measurement of each group of mice, we found that lean ratio in the model group was reduced 4.7%. In contrast, AKG treatment significantly increased the lean ratio of D-gal-induced aging mice by 16.6% (Figure 1C, *p* < 0.001). Next, we analyzed the tibialis anterior (TA) and gastrocnemius (GAS) muscles of the hind limbs from different treatment groups. Compared to the control group, the ratios of TA and GAS in the model group were decreased by 18.2% and 16.1%. AKG intervention restored the ratios of TA and GAS in the model group to the levels of the control group (Figure 1D,E). Behavioral experiments showed that the running time (muscle endurance), maximum exercise speed (muscle explosiveness), and grip strength of mice in the model group were significantly lower than those in the control group, with reductions of 18.4%, 12.8%, and 24.5%, respectively (Figure 1F–H). AKG intervention restored these parameters in the model group to control group levels. Overall, these results indicated that the sarcopenia model was successfully established and AKG intervention could improve skeletal muscle mass and function in D-gal-induced sarcopenia.

### 3.2. Effect of AKG on Structure and Composition of Myofiber in D-Gal-Induced Aging Mice

**Figure 2:**
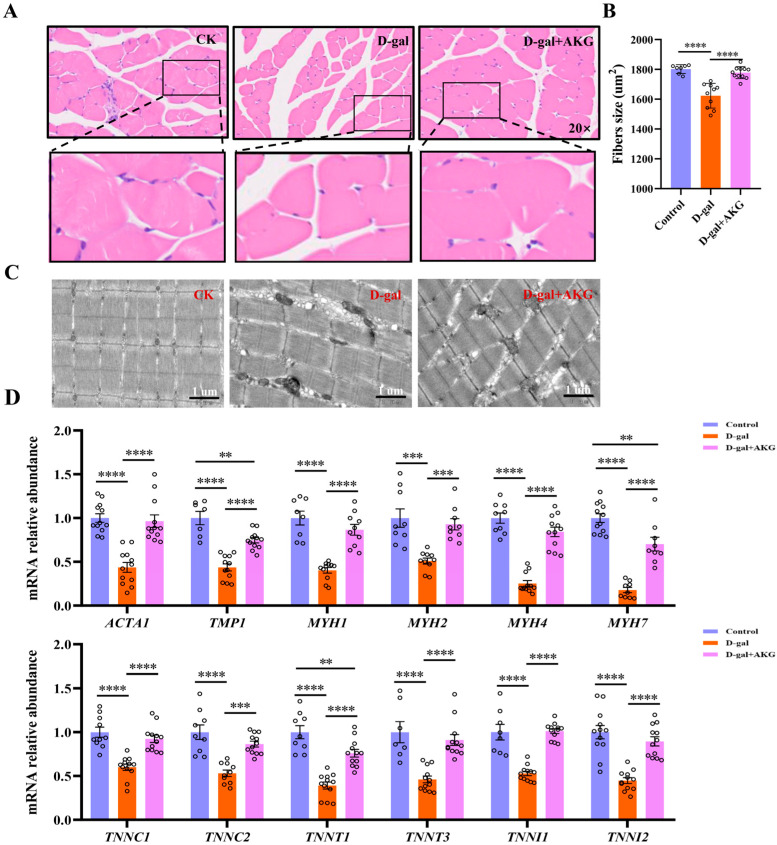
Effect of AKG on the structure and composition of myofiber in D-gal-induced mice. (**A**) Representative H&E staining images of myofibers’ cross-sectional area, (*n* = 3 mice/group). (**B**) The mean cross-sectional area of muscle fibers was analyzed by randomly selecting five representative regions from each sample, comprising approximately 300 muscle fibers. (**C**) Representative images of the ultrastructure of muscle fibers, (*n* = 3 mice/group). (**D**) Relative expression of ACTA1, TMP1, TNNC1, TNNC2, TNNT1, TNNT3, TNNI1, TNNI2, MYH1, MYH2, MYH4, and MYH7 mRNA, (*n* = 6 mice/group). ** *p* < 0.01, *** *p* < 0.001, **** *p* < 0.0001.

As age increases, the loss of muscle mass is characterized pathologically by a decrease in the number of muscle fibers or a reduction in their cross-sectional area [8]. We performed H&E staining on skeletal muscle fibers from different groups, with results shown in Figure 2A,B. Compared to the control group, the cross-sectional area of skeletal muscle fibers in the model group was obviously reduced about 10%, and the muscle fibers became loosely arranged, indicating an increase in the connective tissue between muscle bundles or a decline in the structural integrity of the muscle fibers themselves. However, AKG treatment restored the cross-sectional area of muscle fibers in the model group to levels comparable to the control group. We further observed the ultrastructure of the muscle fibers using transmission electron microscopys (TEMs), as shown in Figure 2C. Myofibers in the control group exhibited a tight and organized arrangement of myofibrils with clear boundaries of the light band, dark band, Z line, and M line. In contrast, the muscle fiber structure in the model group was significantly disrupted, characterized by the loss of periodic features within the sarcomeres, indistinguishable I bands, A bands, Z lines, and M lines, as well as disordered thick and thin filament arrangements, accompanied by abnormal vacuole formation in the cytoplasm. The muscle fiber ultrastructure in the AKG intervention group was significantly restored, with near-complete integrity of the sarcomeres and a clear structure of I bands, A bands, Z lines, and M lines, resembling the organization of the control group. Next, we measured the mRNA expression levels of key proteins that make up the muscle fiber structure. As shown in Figure 2C, compared to the control group, the mRNA expressions of actin (*ACTA1*), tropomyosin (*TMP1*), troponin complex (*TNNC1*, *TNNC2*, *TNNT3*, *TNNI1*, and *TNNI2*), and myosin heavy chains (*MYH1*, *MYH2*, *MYH4*, and *MYH7*) were significantly reduced to varying degrees in the model group. AKG treatment was able to increase the mRNA expressions of these proteins to varying degrees. In summary, AKG intervention can enhance the synthesis of major structural proteins in the skeletal muscle of D-gal-induced aging mice, thereby improving the structure of muscle fibers.

### 3.3. Effect of AKG on Protein Synthesis and Degradation Signaling Pathways of Myofibers in D-Gal-Induced Aging Mice

**Figure 3:**
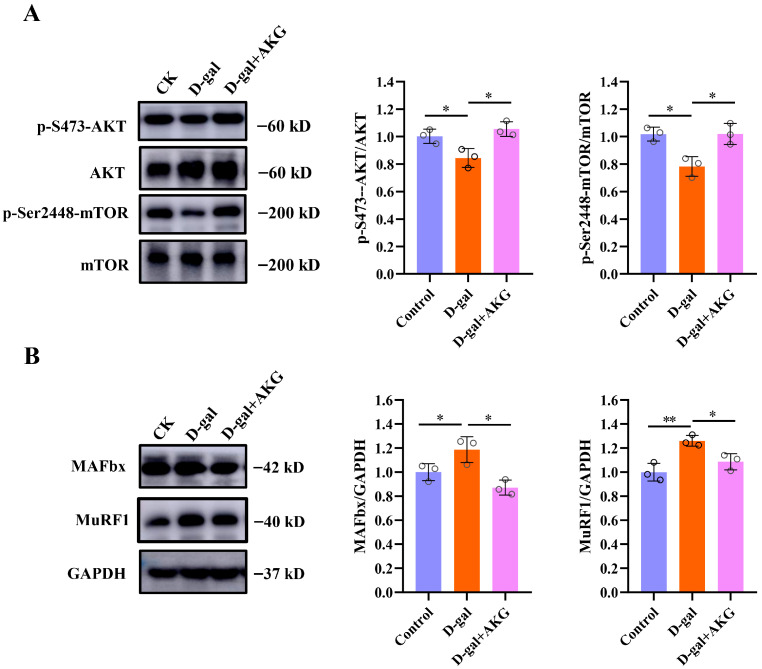
Effect of AKG on protein synthesis and degradation signaling pathways of myofibers in D-gal-induced aging mice. (**A**) Western Blot analysis of protein expression levels of p-S473-AKT, AKT, p-Ser2448-mTOR, mTOR (*n* = 3 mice/group). (**B**) Western Blot analysis of protein expression levels of MAFbx, MuRF1 and GAPDH, (*n* = 3 mice/group). * *p* < 0.05, ** *p* < 0.01.

Protein homeostasis balances the size of myofibers by regulating the processes of protein synthesis and degradation in response to anabolic and stress signals [26]. Therefore, we measured the anabolic and catabolic signals in the skeletal muscles of mice from different groups. Compared to the control group, the ratio of p-S473-AKT/AKT, p-Ser2448-mTOR/mTOR was significantly downregulated in the model group (*p* < 0.05), with decreases of 22.0% and 18.2%, respectively. This indicates that the mTOR/AKT pathway is inhibited, suggesting that the protein synthesis pathway is suppressed. However, AKG intervention significantly alleviated the suppression of the protein synthesis pathway in the skeletal muscles of D-gal-induced aging mice (as shown in Figure 3A). Subsequently, we examined the protein degradation pathways in the skeletal muscles of different groups, with results shown in Figure 3B. Compared to the control group, the protein expression levels of muscle-specific atrophy enzyme MAFbx and MuRF1 were significantly upregulated in the model group, with increases of 12.0% and 26.1%, respectively. AKG intervention significantly downregulated the expression of these two proteins to normal level, indicating that AKG treatment can inhibit the degradation of myofiber proteins.

### 3.4. Effect of AKG on Thermogenesis and Mitochondrial Functionin D-Gal-Induced Aging Mice

**Figure 4:**
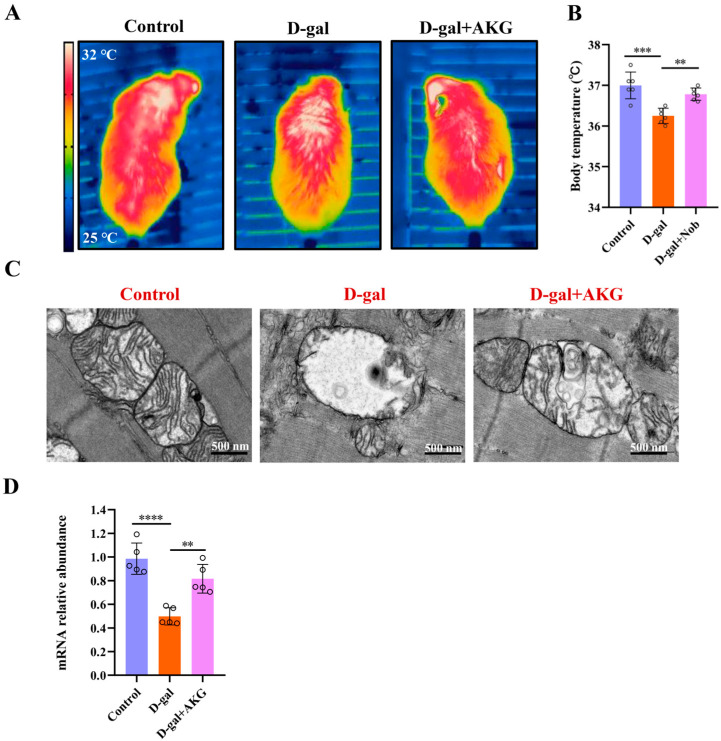
Effect of AKG on thermogenesis and mitochondrial function in D-gal-induced aging mice. (**A**) Representative infrared thermal image map, the gradation of the color of the temperature scale on the left, from blue to red, represents a gradual increase in temperature from 25 °C to 32 °C. (**B**) Mean body surface temperature, (*n* = 6 mice/group). (**C**) Representative images of mitochondrial ultrastructure, (*n* = 3 mice/group). (**D**) Relative expression of *COX1*, (*n* = 6/group). ** *p* < 0.01, *** *p* < 0.001, **** *p* < 0.0001.

Skeletal muscles not only support physical functions such as exercise but also provide energy and maintain body temperature [27]. Subsequently, this study measured the temperature changes in three groups of mice after acute cold exposure using infrared thermography. The control group exhibited extensive red areas (indicating higher temperatures) on their dorsal surfaces (Figure 4A). In contrast, the D-gal group showed significantly reduced red coloration compared to the control group. Notably, the AKG intervention group demonstrated a marked increase in red areas on the dorsal region (Figure 4A). Mitochondria are where most of the cell’s energy is produced [28]. Aging mitochondria have an increased abnormal rate [29,30], characterized by swelling, enlargement, and damage to both morphology and structure [31]. In the control group, the structure of mitochondria in myofibers was normal, and cristae could be clearly seen. (Figure 4C). However, the D-gal group had obvious mitochondrial swelling with partial loss of cristae (Figure 4C). Moreover, AKG treatment significantly alleviated the mitochondrial ultrastructure damage in the myofibers (Figure 4C). *COX1* is the first subunit of cytochrome C oxidase, a key protein complex in the mitochondrial respiratory chain. This complex is responsible for the final step of cellular aerobic respiration and is crucial for the production of energy (ATP). Therefore, we assessed the mRNA expression of the *COX1* gene and found that the expression of *COX1* in the model group was reduced by 49.47% compared to the control group (Figure 4D). Notably, AKG intervention was able to restore *COX1* expression to the level of the control group.

### 3.5. Effect of AKG on Antioxidant and Anti-Inflammatory Abilities in D-Gal-Induced Aging Mice

**Figure 5:**
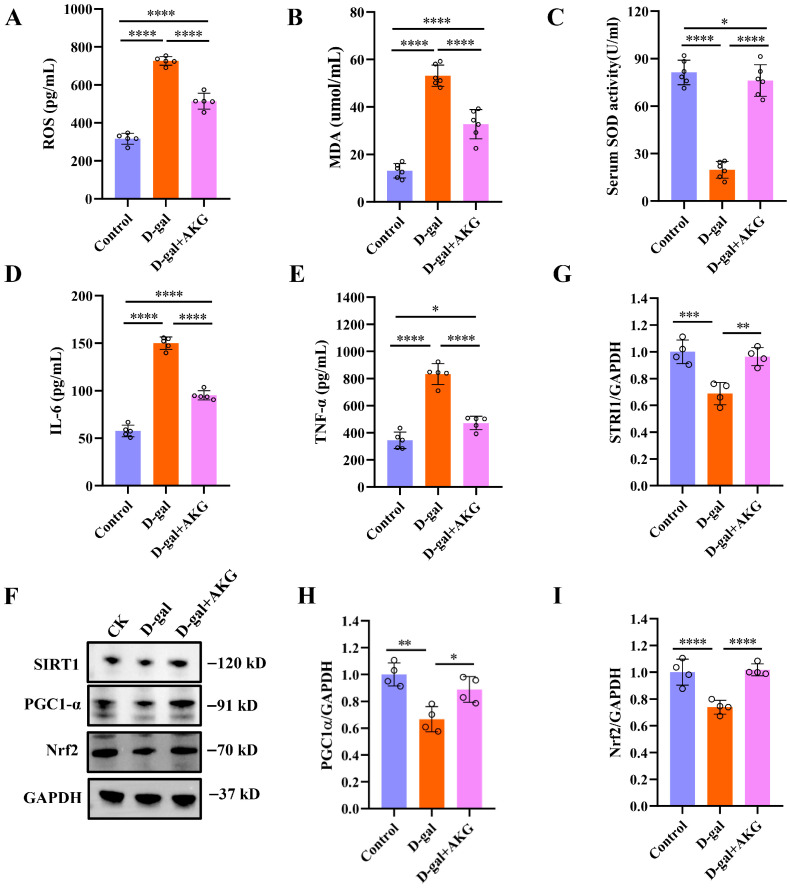
Effect of AKG on antioxidant and anti-inflammatory abilities in D-gal-induced aging mice. (**A**) The content of ROS in serum, (*n* = 5 mice/group). (**B**) The content of MDA in serum, (*n* = 6 mice/group). (**C**) SOD activity in serum, (*n* = 6 mice/group). (**D**) The levels of IL-6 in serum, (*n* = 5 mice/group). (**E**) The levels of TNF-α in serum, (*n* = 5 mice/group). (**F**–**I**) Western Blot analysis of protein expression levels of SIRT1, PGC-1α, Nrf2 and GAPDH, (*n* = 4 mice/group). * *p* < 0.05, ** *p* < 0.01, *** *p* < 0.001, **** *p* < 0.0001.

Mitochondrial respiration not only produces ATP but also generates ROS [21,32]. Oxidative damage caused by the accumulation of ROS is one of the main causes of aging. If antioxidant enzymes in the body cannot promptly eliminate excess ROS, it will lead to mitochondrial dysfunction and accelerated aging. Therefore, we assessed whether AKG can improve the antioxidant capacity in D-gal-induced aging mice. Compared to the control group, serum levels of ROS and malondialdehyde (MDA) in D-gal-induced aging mice increased approximately 1.3 and 4.2 times, respectively, while AKG treatment reduced 29.21% and 38.46% in the model group (Figure 5A,B). Superoxide dismutase (SOD) is a key antioxidant enzyme in the body, serving as a primary substance for scavenging free radicals. Compared to the control group, the activity of SOD in the serum of the model group mice decreased by 78.2%, but AKG intervention increased the SOD activity in model mice by 4.6 times (Figure 5C). Mitochondria are also central regulatory organelles of inflammation, and damaged mitochondria can trigger inflammatory responses [33]. In comparison to the control group, the serum levels of tumor necrosis factor-alpha (TNF-α) and interleukin-6 (IL-6) in the model mice rose by approximately 1.5-fold and 1.6-fold, respectively. However, the intervention with AKG notably reduced the secretion of TNF-α and IL-6 in the serum of the model mice. We next measured the expression of the oxidative stress-related pathway SIRT1/PGC-1α/Nrf2. Although D-gal induction treatment reduced the expression of SIRT1, PGC-1α and Nrf2 of skeletal muscle relative to the control group, AKG treat significantly elevated the expression of SIRT1, PGC-1α and Nrf2 (Figure 5F–I). Together, these results provide evidence for the ability of AKG to modulate antioxidant activity in skeletal muscle, indicating that AKG may have protective effects on oxidative damage in aged mice.

## 4. Discussion

Sarcopenia is a syndrome of aging characterized by a decline in skeletal muscle mass and function, posing significant public health challenges that threaten the independence and quality of life of the elderly population [4,5,34]. The pathological mechanisms underlying sarcopenia are multifactorial, involving dysregulation of muscle protein synthesis and degradation, mitochondrial dysfunction [35], chronic inflammation, and oxidative stress [36,37]. During the aging process, normal cells are subjected to pro-senescent stimuli, such as reactive oxygen species (ROS) or inflammation, exhibit increased expression of senescence markers (e.g., p21, p16, p53). This leads to the accumulation of senescent cells, which produce ROS and the senescence-associated secretory phenotype (SASP) in large amounts, contributing to tissue dysfunction, including skeletal muscle atrophy [38]. Natural aging models are often time-consuming and costly, making accelerated aging models, such as the D-galactose-induced aging model, more favorable due to their ease of application, shorter study duration, and higher survival rates [39]. This model is widely adopted for its convenience and fewer side effects, effectively mimicking the oxidative stress and inflammation associated with sarcopenia [2,3]. Therefore, in this research, we used D-galactose induction to establish the sarcopenia model. In this study, we investigated the effects of α-ketoglutarate (AKG), an intermediate in the Krebs cycle known for its antioxidant properties, on sarcopenia. Our findings indicate that AKG treatment significantly improved muscle mass and functional performance in aged mice. This aligns with findings by Cai et al., who reported AKG effectively attenuated corticosterone-induced protein degradation and rescued the muscle atrophy and dysfunction in a Duchenne muscular dystrophy mouse model [17].

Skeletal muscle, which has high energy demands, is enriched with mitochondria [40]. While mitochondria generate ATP through oxidative phosphorylation, this process also results in excessive ROS production, leading to decreased mitochondrial membrane potential, impaired ATP synthesis, and disrupted calcium homeostasis [41,42]. These changes accelerate myofiber atrophy via the activation of mitochondria-dependent apoptotic pathways [43]. Previous studies have shown that Mfn2 fusion-deficient mice exhibit substantial ROS production and reduced mitochondrial respiration, resulting in significant reductions in muscle cross-sectional area [44]. Thus, oxidative stress emerges as a central driver of skeletal muscle aging [45]. Our research demonstrated that AKG effectively reduced ROS and MDA levels while significantly increasing SOD activity, indicating its role in alleviating oxidative damage by enhancing the antioxidant defense system. Therefore, we focused on the SIRT1/PGC-1α/Nrf2 signaling axis, which plays a central role in regulating mitochondrial quality. SIRT1, an NAD^+^-dependent deacetylase, activates PGC-1α, promoting mitochondrial DNA replication, respiratory chain protein synthesis, and fatty acid oxidation [46,47]. Concurrently, PGC-1α works with Nrf2 to enhance antioxidant defenses [48]. Nrf2 mitigates oxidative damage by inducing the expression of antioxidant enzymes, thereby interrupting the cycle of oxidative stress [49]. Our experimental results revealed that AKG intervention significantly upregulated the expression levels of SIRT1, PGC-1α, and Nrf2 in aged skeletal muscle, accompanied by increased antioxidant enzyme activity. In addition, we observed that AKG intervention significantly reduced pro-inflammatory cytokine levels (e.g., TNF-α, IL-6) in aged skeletal muscle, effectively attenuating proteolytic catabolism. This aligns with findings by Shahmirzadi et al., who reported that AKG can induce IL-10 to inhibit chronic inflammation, thus extending health benefits and lifespan [18].

AKG is a substance naturally produced in the human body, and regulatory agencies have recognized its safety and edibility [15]. However, only at moderate levels can AKG effectively protect organisms from various stressors and pathological states [50]. While this study provides preliminary evidence within an absolutely safe physiological range supporting AKG as a potential intervention for sarcopenia, it is crucial to validate the effective dosage and safety of AKG through further clinical trials. It is important to note that although D-galactose serves as a useful aging model, it may not fully capture the complexity of the human aging process. Additionally, this study focused solely on the D-galactose model without incorporating other aging models, such as naturally aged mice or genetic models, which may limit the broader applicability of the results. Future research should employ a more diverse range of models to further validate and explore these findings, while also considering the expansion of sample size, gender differences, and the synergistic effects with other interventions (such as exercise or combined nutritional strategies) to facilitate clinical translation.

## 5. Conclusions

**Figure 6:**
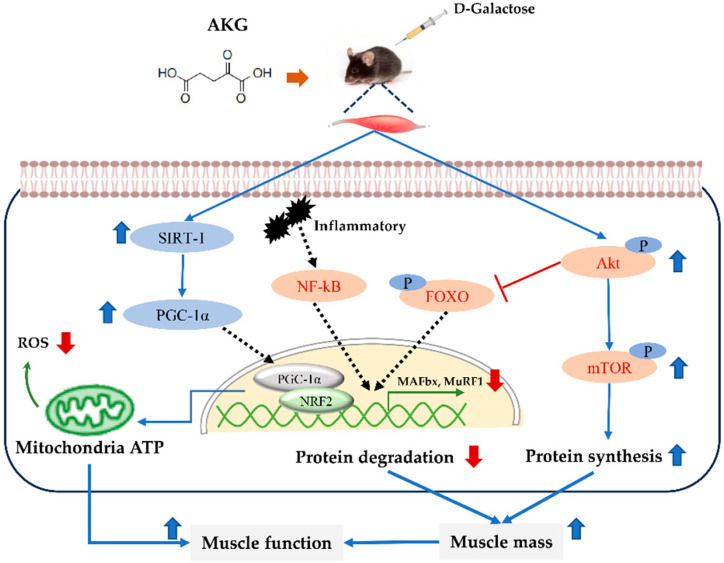
Schematic diagram of AKG-mediated prevention of skeletal muscle atrophy.

In summary, AKG significantly ameliorated muscle mass, exercise endurance, grip strength, and cold tolerance in D-gal-induced aging mice (Figure 6). Also, AKG could regulate protein homeostasis, thereby enhancing the protein composition and size of myofibers in D-gal-induced aging mice. Furthermore, AKG enhanced SOD activity in the skeletal muscle of D-gal-induced aging mice and scavenged reactive oxygen species (ROS) by activating the SIRT1/PGC-1α/Nrf2 pathway, thereby improving mitochondrial function. Consequently, supplementation of AKG can increase muscle mass and enhance muscle function, positioning it as a promising candidate for the prevention and treatment of sarcopenia.

## Figures and Tables

**Table 1:** Primer sequences of RT-qPCR.

Gene Name	Primer Sequence 5′→3′ (Forward)	Primer Sequence 5′→3′ (Reverse)
*ACTA1*	TACCACCGGCATCGTGTTG	GCGCACAATCTCACGTTCAG
*TMP1*	TTGAAAGCCGAGCCCAAAAAG	TCATACTTCCGGTCAGCATCTT
*TNNC1*	GCGGTAGAACAGTTGACAGAG	GACAGAAACTCATCGAAGTCCA
*TNNC2*	GAGGCCAGGTCCTACCTCAG	GGTGCCCAACTCTTTAACGCT
*TNNT1*	ACTAAAAGACCGCATTGGAGG	AGCTCCCATGTTGGACAGAAC
*TNNT3*	ACTGCTCCTAAGATCCCGGAA	ATGAGGTCCTTGTTTTGACGC
*TNNI1*	ATGCCGGAAGTTGAGAGGAAA	TCCGAGAGGTAACGCACCTT
*TNNI2*	CGGAGGGTGCGTATGTCTG	CAGGTCCCGTTCCTTCTCA
*MYH1*	CTCTTCCCGCTTTGGTAAGTT	CAGGACATTTCGATTAGATCCG
*MYH2*	TGGAGGGTGAGGTAGAGAGTG	TTGGATAGATTTGTGTTGGATTG
*MYH4*	CCGCATCTGTAGGAAGGGG	GTGACCGAATTTGTACTGATGT
*MYH7*	CCTGCGGAAGTCTGAGAAGG	CTCGGGACACGATCTTGGC
*COX1*	GAGATGAACAGGGGCACCAA	ATCAGAACGAGCGCAGTGAA
*GAPDH*	TGGCCTTCCGTGTTCCTAC	GAGGCTGTGAAGTCGCA

## Data Availability

The original contributions presented in this study are included in the article. Further inquiries can be directed to the corresponding author.

## Abbreviations

The following abbreviations are used in this manuscript:

AKG	α-ketoglutarate
AKT	Protein kinase B
D-gal	D-galactose
FoxO	Foxhead box O
GAS	Gastrocnemius
IL-6	interleukin-6
MAFbx	Muscle atrophy F-box
MDA	Malondialdehyde
MuRF1	Muscle RING Finger 1
mTOR	Rapamycin
Nrf2	Nuclear factor erythroid 2-related factor 2
PGC-1α	Proliferator-activated Receptor Gamma Co-activator 1-alpha
ROS	Reactive oxygen species
SIRT1	Silent Information Regulator Transcript 1
SOD	Superoxide dismutase D
TA	Tibialis anterior
TEMs	Transmission electron micrographs
TNF-α	tumor necrosis factor-alpha
